# The Asymmetric Emotional Associations to Beverages: An Approach through the Theory of Positive Asymmetry

**DOI:** 10.3390/foods10040794

**Published:** 2021-04-08

**Authors:** Cristina Calvo-Porral, Sergio Rivaroli, Javier Orosa-González

**Affiliations:** 1 Business Department, Facultad Economía y Empresa, University of A Coruña, Campus Elviña s/n, 15001 Coruña, Spain; jorosa@udc.es; 2 Department of Agricultural and Food Sciences, University of Bologna, Viale Giuseppe Fanin 50, 40127 Bologna, Italy; sergio.rivaroli@unibo.it

**Keywords:** beverages, emotions, consumption, Theory of Positive Asymmetry, satisfaction

## Abstract

Consumers experience mainly positive emotions in response to food products, and the reason is that, for most individuals, eating and drinking is a pleasurable experience. On this premise, in light of the Theory of Positive Asymmetry, this study answers the following question: “*What emotions prevail in beverage consumption*?” A MANOVA test was developed comparing emotions associated with spirits (*n* = 247), alcoholic beverages (*n* = 560) and non-alcoholic beverages (*n* = 254). The findings report that the positive asymmetry of emotions occurs in beverage consumption, regardless of the type of beverage product, since pleasant or positive emotions are primarily associated with beverages’ consumption. The analysis suggests that individuals predominantly associate beverages with pleasant emotions, regardless of the type of beverage, while the level of alcohol content seems to be influencing the different emotions and affect. The research results provide valuable insights to help managers and marketers understand the choice and preference for different beverages.

## 1. Introduction

Consumer’s food-evoked and food-related emotions provide valuable information beyond overall liking, and better predict the individuals’ food preference and choice behavior [1]. Consequently, understanding these emotional associations could provide a better knowledge of beverage-related attitudes, preferences and behaviors, such as beverage, choice and intake [2].

Similarly, the Theory of Positive Asymmetry demonstrates a positive bias or a hedonic asymmetry showing that consumers have a predominantly positive affective disposition towards food products. Based on this theory, we can state that foods and beverages elicit mainly positive emotions. However, the idea that positive or pleasant emotions prevail in food products was pointed out by [3] and by [4], since these authors questioned whether negative/unpleasant emotions might not predict and influence consumer behavior like positive ones. Moreover, prior studies have verified and empirically tested the Theory of Positive Asymmetry, but most of these studies have focused on one product category individually, without comparing different product categories [5]. To the best of our knowledge, this is the first study that examines the applicability of this positive emotional bias comparing different beverages with different levels of alcohol content, ranging from spirits to non-alcoholic beverages. On this premise, the aim of this study is twofold: firstly, to analyze whether the Theory of Positive Asymmetry applies to the beverage sector; secondly, to explore the emotional associations concerning the beverage’s alcohol content level considering three drinks’ categories that consumers may choose during social events or convivial situations, such as spirits, beers, and coffee.

Emotions experienced in food consumption and associated with food products will likely determine which foods are selected at the point of purchase, product success and product loyalty [6]. In this context, the research’s major contribution is providing further knowledge about the emotional associations with beverages, differentiating between products from the same product category. The findings will provide a deeper understanding of a product category’s emotional landscape, such as beverages, which can be used to identify new product opportunities to marketers and marketing managers and guide new product development in this sector.

## 2. Literature Review

### 2.1. Emotions in Food and Beverage Consumption

The critical role of emotions in consumer behavior has been widely reported and prior research has examined how the emotional response to food products influences consumer acceptance, attitudes, product choice and purchase intention [7,8,9,10]. However, a broader perspective on how individuals experience beverages and food products is required to consider all associations assigned to products based on experience and learning, and part of these associations are affective associations [1]. Therefore, the analysis of consumers’ emotional associations with food products and emotional responses to foods provides valuable information [11].

In consumer research and applied sensory studies, the term emotion is often used to indicate a brief emotional experience elicited by a product [12]; but there is not a formal definition on the conceptualization of this term. In his seminal work, [13] defined the concept of emotion as “*short-term affective responses to the appraisal of particular stimuli, having reinforcement potential*”. Likewise, emotions could be understood as the conscious subjective experience that accompanies the affective states created by stimuli [13]. Later, [14] conceptualized emotions as the specific response to a stimulus, an object or event, and it can be stated that emotions are a function of multiple information associations underlying the emotional product [15]. On a broad level, emotions can be viewed on two dimensions, namely, positive/pleasant versus negative/unpleasant emotions [11], being that the classification of emotions as positive and negative affect the most popular [10], given that the valence is the primary dimension of emotional meaning [12].

Prior research highlights the close relationship between emotions and food and beverage consumption [11], since the sensory attributes of food products and beverages constitute a source of emotions [16]. In fact, food products can elicit a wide range of emotions associated with the food itself or with the food experience, such as the context in which food is consumed [12,17]. So, food-related emotions could effectively differentiate categories of food products [18].

In this context, previous studies indicate that consumers’ response to a beverage does not only depend on the beverage itself but also on the associated emotions [19]. The reason is that drinking experiences are influenced by two processes: beverage identification through sensory perception and the creation of associations that individuals assign when thinking about a specific beverage [19]. Further, these emotional associations to beverages may arise through three different effects: firstly, how consumers conceptualize the product category; secondly, the individuals’ responses to the sensory attributes and characteristics of a specific product—named as the sensory effect; thirdly, the attitude and disposition of consumers to the product category. Similarly, food names may elicit emotional associations [19] that may result in stronger emotional associations than the food’s sensory experience [20]. Consequently, emotions can be evaluated in response to a food name [3], reflecting the consumer’s memory of the food and his/her past experiences with that product [11,20].

### 2.2. The Theory of Positive Asymmetry

Prior research in consumer behavior has predominantly focused on consumers’ unpleasant or negative emotions, strongly emphasizing the negative emotional states related to food consumption. [16] showed that products evoke emotional responses that are mainly positive, suggesting that positive or pleasant emotions tend to be more relevant and strongly experienced than unpleasant emotions in product consumption. Also, these authors demonstrated that individuals overwhelmingly use positive, rather than negative, words when describing food or beverage consumption experiences. Similarly, individuals use positive terms with a higher frequency related to food products. So, it can be stated that food products mainly elicit positive or pleasant emotions, and when evaluating food products that are not well-liked, consumers tend to use negative emotions sparingly [20].

Accordingly, [16] referred to this positive bias as a “hedonic asymmetry” and developed the Theory of Positive Asymmetry. This theory states that individuals have a predominantly positive affective disposition towards food products and the positive nature of food experiences. Thus, these authors showed that positive or pleasant emotions occur more often than unpleasant ones in food consumption.

Later, [16] tried to explain this phenomenon and suggested that individuals only consume those products that are expected to have a pleasant emotional impact. Likewise, another potential explanation of this positive bias is that food products are designed to be appealing to consumers; thus, eliciting pleasant emotions [16]. This asymmetric emotional bias was empirically tested for food products and confirmed for different product categories [11,21,22], such as chocolate, potato chips [4] or wine [15], extending the theory. In this vein, the authors propose that even though food products are usually associated with positive or pleasant emotions, negative/unpleasant emotions may also occur and may be associated with beverages, motivating the present research.

### 2.3. Research Hypotheses Development

#### 2.3.1. Emotions Associated with Beverages

Relatively few studies exist in the marketing field concerning emotions and beverages consumption, and the existing ones mainly report on positive emotions related to beverages consumption focusing on beer and wine [5,23]. Following [19], an emotional association with a beverage or food product could be defined as “*the emotional connotation that reflects what the product is communicating to the individual*”. Other authors, such as [16], report that food and beverage names evoke associated memories of previous eating and drinking occasions; that is, in turn, linked to emotions experienced on these occasions. Likewise, some authors highlight that negative emotions are important to investigate in beverage consumption since beverages benefit from reducing negative emotions and making consumers feel better [18].

Interestingly, prior research on the topic shows differences in the emotions associated with different types of beverages. For example, [24] demonstrated that spirits and beer are mainly associated with feeling ‘‘euphoric’’, ‘‘happy’’ and “appealing”. Other studies focused on emotions associated with wine consumption, showing that the two emotional terms associated with wine consumption are “feeling elegant” and “feeling interesting”. Similarly, authors like [25] showed that the consumption of wine is associated with low-arousal positive emotions such as pleasure, enjoyment, fulfilment or feeling relaxed. Further, [5] indicated that wine increases pleasant emotions, while there is no evidence that wine decreases negative emotions.

Regarding the consumption of beer, authors like [26] highlighted that beer shows a strong positive emotional profile, being mostly associated with positive high arousal emotional responses. However, the emotions mainly reported to be associated with beer are “enjoyment”, “feeling peaceful”, “adventurous” and “amusing” [27]. Finally, regarding coffee consumption, prior research notes that consumers tend not to have unpleasant feelings in coffee consumption [27]. More precisely, the most relevant emotions associated with coffee are “feeling pleasure”, “feeling energetic”, “feeling active”, “warm”, “comfortable”, and “productive”. Conversely, the negative emotions primarily associated with coffee are “feeling bored”, “disgusted”, “annoyed”, “worried” or “grouchy” [28]. In this context, the research developed by [29] found a set of emotional terms specific to coffee that was not previously found to be relevant to other beverages, such as “staying mentally alert” and “focused”, “motivated”, “feeling productive” and “clear-minded”.

On the other hand, prior studies have reported that non-alcoholic beverages are associated with negative and neutral emotions, such as “feeling rational” or “feeling disappointed” [26]; however, it could be expected that weak positive emotions are associated with non-alcoholic beverages due to the absence of alcohol. On these premises, it is hypothesized that:

**Hypothesis 1** **(H1).**  *Consumers will associate positive emotions rather than negative emotions to beverage consumption.*

#### 2.3.2. Consumer Satisfaction with Beverages

One of the seminal conceptualizations of satisfaction is based on the expectancy-disconfirmation paradigm [30], which supports that consumers form expectations compared to the real product or service performance. So, individuals’ satisfaction judgments entail an evaluative process in which expectations about a product or service are compared with real performance, thus creating satisfaction or dissatisfaction [31]. More precisely, if the product or service fails to meet the customers’ needs or expectations, consumers will respond with negative emotions, while if the product or service is perceived as better than expected, consumers will respond with positive emotions [32]. A vast body of literature reports that emotions are a core component of consumer satisfaction since positive/pleasant emotions and consumer satisfaction are positively linked [33], while negative emotions are associated with negative affect [34,35]. There is a direct relationship between emotions and consumer satisfaction, with the emotions-satisfaction link being valence-congruent [36,37].

Regarding products’ consumption, the marketing literature supports that food products have an emotional impact on consumer satisfaction [20,34]. Thus, it seems coherent to suggest that feelings of pleasure should positively influence satisfaction and that consumers will experience positive emotions rather than negative emotions derived from food products’ consumption [11,16]. Accordingly, the following hypothesis is posed:

**Hypothesis 2** **(H2).**  *Consumers will experience high satisfaction in beverage consumption.*

## 3. Methodology

### 3.1. Sampling and Fieldwork

Various methods have been used to evaluate emotions evoked by food experiences including questionnaires with emotion terms [38,39]. Among these methods, the self-report questionnaires are the most commonly used methods in applied food-related emotion research [12], since the self-reported measure of emotions can capture the “feeling” and emotional aspect of multiple emotional responses. Thus, measuring the consumer emotional response through self-reported emotions is more discriminating than using “liking” measures [1,3]. Accordingly, the research reported here focuses on the emotions associated with beverages, asking participants: “*How do you feel when drinking gin/beer/coffee?*” The reason is that stimuli may include consuming the product and its name [40]. The food product or beverage name elicits a more robust emotional response than product consumption [40].

In the present study, three beverages with different levels of alcohol were selected in order to conduct the research: gin (spirit), beer (alcoholic beverage) and coffee (non-alcoholic beverage). The beverage selection was based on two criteria: we considered the level of alcohol as one relevant factor, and the second criterion applied is the popularity of these beverages among consumers during social events. Beer is one of the most consumed alcoholic beverages worldwide [26], and in terms of alcohol content is positioned between gin and coffee. Similarly, coffee is one of the most consumed beverages and is strongly embedded within social and cultural traditions [28]. Finally, gin has no cultural background in Spain, and it is certainly not as popular as the beer itself, but its presence in the market today shows an interesting development and increasing popularity.

The study involved random sampling, and fieldwork was conducted in January 2020. The participants were captured out through structured online questionnaires among consumers residing in Spain. Three structured questionnaires were prepared, corresponding to each one of the beverages under research. The questionnaires consisted of three sections. In the first section, the participants were asked to evaluate to what extent they experienced the proposed emotions when consuming different beverages. More precisely, the emotions were presented together in the questionnaire, and participants were asked to rate how they felt when consuming the three different beverages on a 7-point intensity scale (“1 = not at all” to “7 = extremely”). Besides, four questions related to customer satisfaction with the beverages were included. The last section of the questionnaire gathered sociodemographic data. Finally, 1278 questionnaires were collected, obtaining 1061 valid responses: spirits (*n* = 247), alcoholic beverages (*n* = 560) and non-alcoholic beverages (*n* = 254), with a confidence level of 95.5%. Regarding the sample description, research participants had to be legally admitted to drinking alcoholic beverage (i.e., at least 18 years old); consequently, the age of participants ranged between 18 and 65, and 69.4% were less than 51 years old. Among them, 40.9% were females, and 77.0% have an academic degree.

### 3.2. Variables and Measurement Scale

The present study has considered 14 positive emotions and 8 negative emotions, as shown in Table 1. The positive emotions included in the measurement scale are adopted from [5], describing the emotional experience associated with beverage consumption; two positive emotions were also included in the scale (i.e., “excited” and “active/alert”). The negative emotions are adopted from [5] and [21]. The emotions examined in the research were split into two categories according to their valence dimension, differentiating between positive/pleasant and negative/unpleasant-emotions. This measurement emotion scale includes approximately the same number of pleasant and unpleasant emotions, which may help analyze the Theory of Positive Asymmetry better. Finally, to measure consumer satisfaction with the beverage product, a 4-item scale was adapted from [31] (Table 1).

## 4. Results

### 4.1. Positive Emotions Associated with Beverages

Mean scores and standard deviations for all emotional categories were calculated. Table 2 shows the mean positive/pleasant emotion values per beverage product. It can be stated that pleasant emotions associated with beverages have mean values ranging from 2.98 or higher for non-alcoholic beverages (Mean_non-alcoholic_ = 4.22) and alcoholic beverage (Mean_alcoholic_ = 4.04); whereas mean values are slightly higher for spirits (Mean_spirits_ = 4.28). Interestingly, the positive emotions more strongly associated with beverages are EMO1 “I feel funny” (Mean_spirits_ = 5.20; Mean_alcoholic_ = 4.69; Mean_non-alcoholic_ = 4.73); EMO4 “I feel happy” for spirits (Mean_spirits_ = 4.64); and EMO12 “I feel pleased/comfortable” for alcoholic and non-alcoholic beverages (Mean_alcoholic_ = 4.46; Mean_non-alcoholic_ = 4.85), suggesting that different types of beverages are associated with different kind of emotions. Conversely, the pleasant emotions more weakly associated with beverages are EMO13 “I feel excited” for spirits (Mean_spirits_ = 3.14); EMO14 “I feel active/alert “for alcoholic beverages (Mean_alcoholic_ = 3.05) and EMO11 “I feel relaxed/calm” for non-alcoholic beverages (Mean_non-alcoholic_ = 2.98); thus, reporting differences related to the type of beverage.

So, our results indicate that different beverages are associated with different emotions or emotional affect, being the spirits the beverage associated with the highest positive emotions. This finding could indicate that the hedonic nature of the beverage product may influence the level of the positive emotions associated with the product and the type of emotional affect.

**Figure 1:**
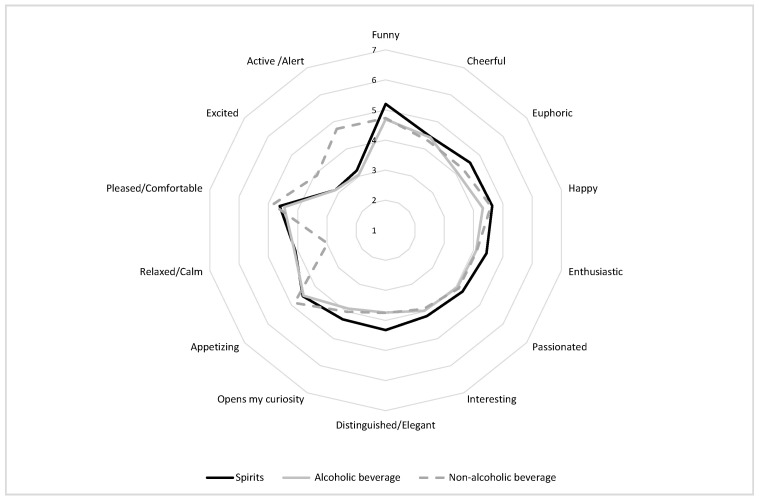
Positive emotional associations with beverage products.

Additionally, the spider plot (Figure 1) shows comparable emotion patterns for the different beverages under analysis, as well as differences in the intensity of the positive emotions associated with beverage consumption, being the spirits the beverage associated with stronger pleasant emotions, followed by non-alcoholic and alcoholic beverages.

### 4.2. Negative Emotions Associated with Beverages

The negative emotions associated with beverage consumption all scored between 1.20 and 2.17, and some of the negative emotions are at the very bottom of mean values (Table 3). Our findings report that the lowest mean score values for the non-alcoholic beverage, while negative or unpleasant emotions were more strongly associated with alcoholic beverages. Results indicate that alcoholic beverages evoke higher negative emotions (Mean_spirits_ = 1.68; Mean_alcoholic_ = 1.68), compared to the non-alcoholic beverage, that evokes the lower negative emotions (Mean_non-alcoholic_ = 1.34). This finding may suggest that the beverage products’ hedonic nature could be the critical factor determining the negative emotions associated with consumption. On the other hand, regarding the negative emotions more strongly associated with beverages, our findings indicate that NEG2 “I feel superior to others” (Mean_spirits_ = 2.17) and NEG4 “I feel bored” (Mean_alcoholic_ = 1.92; Mean_non-alcoholic_ = 1.48) is the stepwise order. Similarly, the unpleasant emotions more weakly associated with beverage consumption are NEG8 “I feel worried” (Mean_spirits_ = 1.44; Mean_alcoholic_ = 1.38; Mean_non-alcoholic_ = 1.20), and NEG7 “I feel tense/nervous” (Mean_spirits_ = 1.46; Mean_alcoholic_ = 1.42; Mean_non-alcoholic_ = 1.28). The findings show that the mean values for the negative/unpleasant emotions are much lower than the mean scores for positive emotions for all the beverages under analysis.

**Figure 2:**
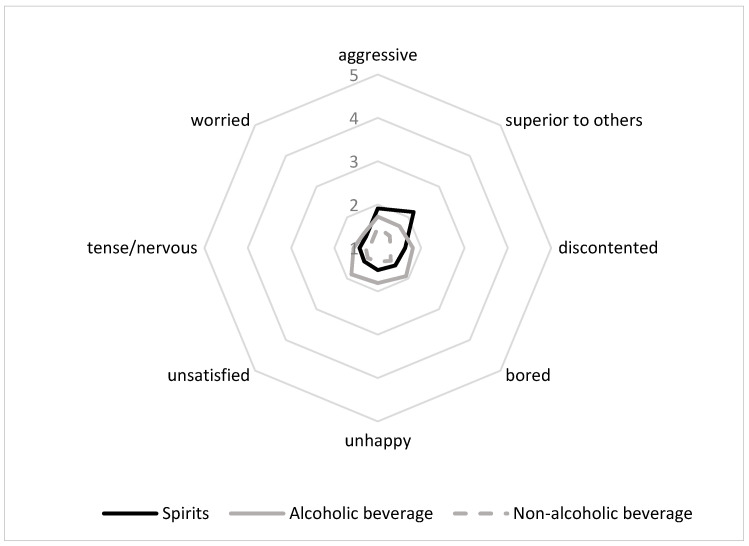
Negative emotional associations with beverage products.

Figure 2 shows comparable negative emotion patterns for the three beverages under analysis, being the non-alcoholic beverage the product associated with lower negative emotions. According to our initial expectations, research findings indicate that consumers mostly associate positive/pleasant, rather than negative/unpleasant emotions to beverages; thus, demonstrating that the Theory of Positive Asymmetry could be applied to beverage products, regardless of the type of beverage and regardless the level of alcohol content.

### 4.3. Satisfaction with Beverage Consumption

Our findings show that all consumers seem to be moderately satisfied with beverages’ consumption, regardless of the type of beverage, since satisfaction mean scores were relatively high for the three beverages analyzed. Likewise, research findings indicate that the level of consumer satisfaction could be related to the level of alcohol content, given that the highest levels of satisfaction are achieved by the non-alcoholic beverage (Mean_non-alcoholic_ = 5.16), followed by the alcoholic beverage (Mean_alcoholic_ = 4.77) and the spirits (Mean_spirits_ = 4.76), as shown in Table 4. Additionally, these results may suggest that consumers could tolerate unpleasant emotions and negative affect to some extent. Despite the association of beverages with slight negative emotions, this is not transformed into a low level of satisfaction with the product.

### 4.4. Multivariate Analysis of Variance

The relationships between the type of beverage and the emotions associated with its consumption were tested through multivariate analysis of variance (MANOVA) using SPSS 18.0 software. Our results indicate that the overall multivariate tests are statistically significant for the three types of beverages under analysis, revealing significant (*p* ≤ 0.05) differences in the emotions associated with beverage consumption [41]. More precisely, the multivariate test was developed using Pillai’s Trace (0.484 (F (52, 12.691), *p* = 0.000) Wilk’s Lambda (0.570 (F (52, 12.917), *p* = 0.000) and Hotelling Trace (0.662, F ((52, 13.144), *p* = 0.000) values. Finally, Tukey post hoc tests were developed to examine significant differences in the mean values of emotions associated with beverages.

#### 4.4.1. Positive Emotions

The positive or pleasant emotions associated with beverages differed significantly among beverages on nine out of fourteen emotion categories (Table 5). The MANOVA results reported significant differences for emotions such as “funny”, “euphoric”, “distinguished/elegant”; “opens my curiosity”; “appetizing”; “relaxed/calm”; “pleased/comfortable”; “excited” and “active/alert”. Conversely, research findings do not report significant differences for the emotions “cheerful”, “passionated,” and “interesting” among the different beverages under analysis. The results show that spirits score higher for positive emotions, followed by non-alcoholic beverage and alcoholic drink, respectively. So, spirits, non-alcoholic and alcoholic is the stepwise order for the positive emotions associated with beverages. Interestingly, positive emotions were more strongly associated with the non-alcoholic beverage for the emotion categories relaxed/calm; excited and active/alert. One potential explanation for these results is that the non-alcoholic beverage selected for the study is coffee.

#### 4.4.2. Negative Emotions

Findings show that the negative emotional affect and its relationship with the types of beverages under analysis are difficult to pinpoint. On the one hand, the MANOVA analysis shows significant differences between negative emotions associated with the beverages examined (Table 6). More precisely, it can be stated that non-alcoholic, alcoholic and spirits are the stepwise order for the strength of negative emotional associations, except for the emotion categories feeling “bored”, “unhappy”, and “unsatisfied” that were more strongly associated with the alcoholic beverage compared with spirits and the non-alcoholic beverage. Interestingly, the spirits received significantly higher scores for negative emotions than alcoholic and non-alcoholic beverages. These results suggest that the alcohol content could be influencing the association with unpleasant emotions or negative affect, and further, the emotions associated with beverage consumption seem to be influenced by the alcohol content.

Finally, and considering that consumers mostly associate positive or pleasant emotions with beverage consumption, the proposed research hypothesis “*H1: Consumers will associate positive emotions, rather than negative emotions to beverage consumption*” is supported; thus, confirming the Theory of Positive Asymmetry for beverages.

#### 4.4.3. Satisfaction

The multivariate analysis and the post hoc Tukey test conducted for the three types of beverages show significant differences in consumers’ satisfaction with beverages (Table 7). More specifically, our findings report that consumers feel higher satisfaction with non-alcoholic beverages. Higher positive emotions associated with the beverage do not drive a greater level of satisfaction; since the higher satisfaction level is achieved by coffee. Besides, it can be stated that the level of alcohol content does not positively impact consumer satisfaction, and maybe other factors of beverage consumption—such as consumption frequency, convenience or utilitarian value—could be influencing consumption satisfaction. Considering the mean values obtained for satisfaction items for the three beverages examined that surpass the threshold of 4.75 for the three beverage categories, it can be stated that consumers feel high satisfaction with beverages’ consumption, supporting the proposed research hypothesis “*H2: Consumers will experience high satisfaction in beverage consumption*”.

## 5. Conclusions

This research empirically tests the Theory of Positive Asymmetry [22], which states that emotional responses elicited or evoked in food products’ consumption are predominantly positive. More precisely, this study tests this emotional bias in beverage consumption, comparing different products in this category, ranging from spirits to non-alcoholic beverages.

The main findings are twofold. In the first place, our study confirms that consumers mostly associate positive or pleasant emotions with beverage products confirming the Theory of Positive Asymmetry [22] in this product category and referring to our specific respondents’ profile. According to our initial expectations, beverages’ emotions are primarily positive, regardless of the beverage product type. So, our findings report that pleasant and positive emotions are associated with beverages more strongly than unpleasant or negative emotions, showing that the Theory of Positive Asymmetry [22] could be applied to all types of beverages. In other words, consumers associate more positive/pleasant than negative/unpleasant emotions with beverages. Secondly, our research reports that beverage-associated emotions are different between products from the same product category. The beverages under analysis showed different emotional associations in terms of valence dimension—pleasant/unpleasant. More precisely, our findings report that spirits are associated with the highest positive emotions compared to alcoholic and non-alcoholic beverages. One potential explanation for this result is that the strong hedonic value of spirits might explain the strong positive emotions associated with their consumption. Interestingly, our research also highlights that consumers’ experience more significant satisfaction in consuming non-alcoholic beverages.

So, the major contribution derived from our research is that consumers predominantly associate beverages with positive/pleasant emotions, regardless of the type of beverage, while the level of alcohol content seems to be influencing the different emotions and affect. Thus, food marketing managers should consider the emotions associated with beverages consumption. The reason is that the food and beverage emotional associations play a crucial role in food products and beverage decisions through the emotional consequences of products’ purchase and consumption [6]. Besides, considering the elasticity of some beverages’ demand and the low switching costs to alternative beverages—such as, for example, switching from beer to spirits—this study helps to understand the rationale behind consumers’ choice and preferences for different beverages, which is grounded on the emotions associated with beverages. The value of this research is showing that the measurement and evaluation of emotions can provide an interesting way of describing food products and beverages. Further, beverages could be labelled by the emotions they are associated with. Finally, and according to [12], the sensory profile of a product could be also modified to increase specific positive emotions or to decrease negative affect.

Nonetheless, this research entails some limitations that provide avenues for future research. Firstly, the inclusion of moderate alcohol consumers could generate different results, given that these consumers may associate different emotions and emotional effect on alcoholic and non-alcoholic beverages. Furthermore, because drinking alcohol can be reinforcing for certain people in certain circumstances (e.g., those who struggle with general coping skills and with social skills deficits), it seems helpful in future works to address how these benefits can be derived from healthier activities [42]. Secondly, future studies could consider the drinking context to analyze the emotions associated with beverages in various consumption places and consider that self-report measures can lead to positive bias because respondents tend to give more expected answers than the real ones. Thirdly, further studies should consider that the emotions associated with beverages may not only depend on the beverage itself but also on the specific individual consuming it, and the individual factor was not considered in the present study. Finally, this study was developed in one market, and for this reason, the research could be replicated in other markets. Addressing these limitations in future research will provide a deeper understanding of beverage consumption.

## Figures and Tables

**Table 1:** Variables and indicators.

Variables	Indicators
Positive emotions Ferrarini et al. (2010)	EMO1: I feel funny
	EMO2: I feel cheerful
	EMO3: I feel euphoric
	EMO4: I feel happy
	EMO5: I feel enthusiastic
	EMO6: I feel passionated
	EMO7: I feel interesting
	EMO8: I feel distinguished/elegant
	EMO9: Opens my curiosity
	EMO10: I feel appetizing
	EMO11: I feel relaxed/calm
	EMO12: I feel pleased/comfortable
	EMO13: I feel excited
	EMO14: I feel active/alert
Negative emotions Laros and Steenkamp (2005); Ferrarini et al. (2010)	NEG1: I feel aggressive
	NEG2: I feel superior to others
	NEG3: I feel discontented
	NEG4: I feel bored
	NEG5: I feel unhappy
	NEG6: I feel unsatisfied
	NEG7: I feel tense/nervous
	NEG8: I feel worried
Satisfaction Oliver (1997)	SAT1: I’m satisfied with this beverage
	SAT2: This beverage meets my needs perfectly
	SAT3: This beverage meets my expectations and desires
	SAT4: This beverage provides me exactly what I need

**Table 2:** Descriptive measures for positive emotions.

Items	Spirits		Alcoholic Beverage		Non-Alcoholic Beverage	
	Mean	SD	Mean	SD	Mean	SD
EMO1: I feel funny	5.20	1.664	4.69	2.015	4.73	2.21
EMO2: I feel cheerful	4.45	1.807	4.42	1.942	4.29	2.087
EMO3: I feel euphoric	4.60	1.810	4.06	2.077	4.28	2.133
EMO4: I feel happy	4.64	1.750	4.32	2.021	4.59	2.126
EMO5: I feel enthusiastic	4.44	1.844	4.09	2.042	4.11	2.109
EMO6: I feel passionated	4.27	1.884	4.05	2.059	4.11	2.019
EMO7: I feel interesting	4.17	1.859	3.97	2.135	3.91	2.219
EMO8: I feel distinguished/elegant	4.32	1.950	3.74	2.069	3.75	2.180
EMO9: Opens my curiosity	4.29	1.849	3.89	2.011	4.01	2.093
EMO10: I feel appetizing	4.52	1.814	4.49	1.959	4.88	1.914
EMO11: I feel relaxed/calm	4.08	1.868	4.11	1.976	2.98	2.109
EMO12: I feel pleased/comfortable	4.61	1.740	4.46	2.016	4.85	1.913
EMO13: I feel excited	3.14	1.798	3.14	1.906	3.92	2.331
EMO14: I feel active/alert	3.21	1.873	3.05	1.879	4.74	2.187
**Average values**	**4.28**	**1.822**	**4.03**	**2.007**	**4.22**	**2.117**

**Table 3:** Descriptive measures for negative emotions.

Items	Spirits		Alcoholic Beverage		Non-Alcoholic Beverage	
	Mean	SD	Mean	SD	Mean	SD
NEG1: I feel aggressive	1.91	1.472	1.72	1.344	1.42	1.198
NEG2: I feel superior to others	2.17	1.730	1.71	1.355	1.39	0.999
NEG3: I feel discontented	1.81	1.203	1.63	1.561	1.29	0.970
NEG4: I feel bored	1.57	1.314	1.92	1.748	1.48	1.192
NEG5: I feel unhappy	1.51	1.165	1.81	1.670	1.32	1.098
NEG6: I feel unsatisfied	1.56	1.170	1.86	1.749	1.33	1.114
NEG7: I feel tense/nervous	1.46	1.101	1.42	1.208	1.28	0.891
NEG8: I feel worried	1.44	1.082	1.38	1.087	1.20	0.796
**Average values**	**1.68**	**1.279**	**1.68**	**1.465**	**1.34**	**1.032**

**Table 4:** Descriptive measures for satisfaction.

Items	Spirits		Alcoholic Beverage		Non-Alcoholic Beverage	
	Mean	SD	Mean	SD	Mean	SD
SAT1: This beverage makes me satisfied	5.15	1.493	4.94	1.765	5.35	1.617
SAT2: This beverage meets my needs perfectly	4.43	1.651	4.73	1.834	4.89	1.768
SAT3: This beverage meets my expectations and desires	4.87	1.761	4.85	1.863	5.18	1.733
SAT4: This beverage gives me exactly what I need	4.58	1.801	4.57	2.057	5.24	1.926
**Average values**	**4.76**	**1.676**	**4.77**	**1.879**	**5.16**	**1.761**

**Table 5:** Univariate tests and comparisons for positive emotions.

Indicators	Type III Sum of Squares	Square Mean	F-Value	*p*-Value	Tukey Post Hoc Test
EMO1: I feel funny	47.572	23.786	5.092	0.003	0.000 * s-a;s-n
EMO2: I feel cheerful	5.270	2.635	0.695	0.500	0.534
EMO3: I feel euphoric	48.923	24.462	5.924	0.003	0.000 * s-a
EMO4: I feel happy	22.440	11.220	2.839	0.049	0.000 * s-a
EMO5: I feel enthusiastic	22.320	11.160	2.743	0.045	0.000 * s-a
EMO6: I feel passionated	8.129	4.065	0.963	0.382	0.388
EMO7: I feel interesting	9.356	4.678	1.066	0.345	0.294
EMO8: I feel distinguished/elegant	60.825	30.413	7.103	0.001	0.000 * s-a;s-n
EMO9: Opens my curiosity	27.247	13.624	3.405	0.034	0.000 * s-a
EMO10: I feel appetizing	26.479	13.239	3.608	0.027	0.000 * s-n;a-n
EMO11: I feel relaxed/calm	256.322	128.161	32.462	0.000	0.000 * s-n;a-n
EMO12: I feel pleased/comfortable	32.667	16.333	4.382	0.013	0.000 * a-n
EMO13: I feel excited	117.846	58.923	14.835	0.000	0.000 * s-a;s-n;a-n
EMO14: I feel active/alert	525.410	262.705	68.665	0.000	0.000 * s-n;a-n
Degrees of freedom= 2					

NOTE: * indicates significant differences between the means of the positive emotion terms elicited by the three beverages (s: spirits; a: alcoholic beverage; n: non-alcoholic beverage) according to Tukey post hoc test.

**Table 6:** Univariate tests and comparisons for negative emotions.

Indicators	Type III Sum of Squares	Square Mean	F-Value	*p*-Value	Tukey Post Hoc Test
NEG1: I feel aggressive	24.235	12.118	6.725	0.001	0.000 * s-n; a-n
NEG2: I feel superior to others	76.347	38.173	20.044	0.000	0.000 * s-a;s-n;a-n
NEG3: I feel discontented	46.929	23.465	12.695	0.000	0.000 * s-n; a-n
NEG4: I feel bored	50.573	25.286	10.738	0.000	0.000 * s-a;a-n
NEG5: I feel unhappy	46.521	23.460	11.195	0.000	0.000 * s-a;a-n
NEG6: I feel unsatisfied	61.868	30.934	13.865	0.000	0.000 * s-a;a-n
NEG7: I feel tense/nervous	14.571	7.285	5.862	0.003	0.000 * a-n
NEG8: I feel worried	11.684	5.842	5.572	0.004	0.000 * a-n
Degrees of freedom = 2					

NOTE: * indicates significant differences between the means of the negative emotion terms elicited by the three beverages (s: spirits; a: alcoholic beverage; n: non-alcoholic beverage) according to Tukey post hoc test.

**Table 7:** Univariate tests and comparisons for satisfaction.

Indicators	Type III Sum of Squares	Square Mean	F-Value	*p*-Value	Tukey Post Hoc Test
SAT1: This beverage makes me satisfied	30.284	15.142	5.429	0.005	0.000 * a-n
SAT2: This beverage meets my needs perfectly	27.124	13.562	4.292	0.014	0.000 * s-a;s-n
SAT3: This beverage meets my expectations and desires	20.755	10.378	3.171	0.042	0.000 * a-n
SAT4: This beverage gives me exactly what I need	85.168	42.584	10.985	0.000	0.000 * s-a;s-n;a-n
Degrees of freedom= 2					

NOTE: * indicates significant differences between the means of satisfaction elicited by the three beverages (s: spirits; a: alcoholic beverage; n: non-alcoholic beverage) according to Tukey post hoc test.

## Data Availability

Research data will be available on request.
